# Balancing deterministic and stochastic assembly during needle aging shapes phyllosphere microbial community complexity and stability

**DOI:** 10.1186/s40793-026-00922-7

**Published:** 2026-07-01

**Authors:** Bing Li, Mingyang Fu, Guangze Jin, Fuqiang Song, Zhili Liu, Nicolas Fanin

**Affiliations:** 1 School of Ecology, Northeast Forestry University, Harbin, 150040 China; 2 Key Laboratory of Sustainable Forest Ecosystem Management-Ministry of Education, Northeast Forestry University, Harbin, 150040 China; 3 Northeast Asia Biodiversity Research Center, Northeast Forestry University, Harbin, 150040 China; 4 College of Life Sciences, Heilongjiang University, Harbin, 150080 China; 5 INRAE, Bordeaux Sciences Agro, UMR 1391 ISPA, Villenave-d’Ornon, F- 33882 France

**Keywords:** Community assembly, Microbial network, Needle age, Needle trait, Phyllosphere microorganism, Evergreen conifer

## Abstract

Phyllosphere microorganisms on leaf surfaces play key roles in maintaining plant productivity and health. The composition of phyllosphere microbial communities may vary during leaf aging, leading to changes in the complexity and stability of these communities. However, the ecological mechanisms underlying these varistions, particularly the relative contributions of deterministic (i.e., environmental filtering) and stochastic assembly processes (i.e., random dispersal and ecological drift) in shaping community dynamics, remain unclear.

In this study, we collected 252 needle samples corresponding to three age cohorts from three representative evergreen coniferous species in natural mixed broadleaved-Korean pine forests throughout Northeast China. Needle traits were determined, and both phyllosphere epiphytic bacterial and fungal communities were assessed using amplicon sequencing.

Our results showed that stochastic assembly dominated microbial communities, and the longer immigration periods contributed to increased microbial diversity and network complexity across needle age cohorts. Deterministic assembly became stronger for both bacterial and fungal communities during needle aging, which was closely related to the increased concentrations of secondary metabolites such as flavonoids, resulting in gradually elevated community similarity. Microbial network stability decreased in older needles, primarily due to the lower modularity and fewer negative interactions among species.

Our study provides comprehensive empirical evidence on the succession of phyllosphere bacterial and fungal communities across needle age cohorts, emphasizing the importance of a dynamic balance between deterministic assembly and stochastic assembly in shaping microbial communities during needle aging.

**Supplementary Information:**

The online version contains supplementary material available at https://doi.org/10.1186/s40793-026-00922-7.

## Introduction

Leaf surfaces provide a widespread and unique habitat for diverse microbial communities in terrestrial ecosystems, collectively referred to as the ‘phyllosphere’ [37]. Phyllosphere microorganisms play crucial ecological roles in maintaining host fitness by regulating nutrient cycling and enhancing pathogen resistance [38, 70]. Previous researches have shown that plant-associated microbial communities are shaped by a combination of stochastic and deterministic assembly processes [31, 62]. For example, stochastic processes usually dominate the establishment of initial microbial communities in the phyllosphere, and the random influxes of new microorganisms are particularly common during this period [34]. Moreover, phyllosphere microbial communities are also regulated by leaf traits [79], which determine the availability of resources such as carbohydrates, nutrients, and secondary metabolites, thereby influencing the survival and activity of microorganisms [67]. Recently, needle age has been recognized as an important factor determining needle traits of evergreen coniferous species [36], and mature needles usually exhibit higher leaf dry matter content but lower leaf nitrogen content than young needles [2, 45]. As a result, phyllosphere bacterial and fungal communities may differ significantly across needle age cohorts, with species richness increasing consistently with needle aging [72]. However, knowledge gaps still remain regarding how assembly processes vary and shape phyllosphere microbial communities as needles age.

Understanding the emerging properties of microbial networks, beyond diversity and composition, is increasingly recognized as a powerful tool for investigating the successions of microbial communities [15]. This reflects the fact that microbial communities are not random combinations of species, but rather structured and complex ecological networks with multiple cooperative (e.g., cross-feeding) and competitive interactions (e.g., nutrient competition) [24]. Complexity is a core network feature, and higher network complexity (i.e., more species and stronger interspecific interactions) contributes to the enhanced community-level capabilities in decomposition, phosphorus mineralization, and carbon uptake [51, 71]. Network complexity is usually positively influenced by biodiversity [4], notably because increasing the number of taxa provides more opportunities for establishing additional associations among community members [42]. On the contrary, higher environmental stresses such as carbon or nitrogen limitation may act as filters against fast-growing microbial taxa, resulting in simpler networks [58]. As such, variations in leaf traits may affect the network complexity of phyllosphere microbial communities [80], but how it changes across needle age cohorts with different patterns in microbial diversity and needle traits remains unexplored.

Building on this, microbial network stability (i.e., the ability of microbial communities to resist environmental fluctuations) often contributes to maintaining the productivity of plant communities and ecosystem multifunctionality [6, 84]. Recent studies suggest that higher species richness and network complexity enhance the resistance of microbial networks to environmental disturbances such as drought and nutrient limitation, notably because they increase the abundance of stress-tolerant species and foster compensatory dynamics among species via resource transfer [59]. However, other studies indicate that stable networks are not necessarily complex, meaning that network stability depends not only on the community size but also on network architecture [18, 78]. In particular, modular networks with more negative interspecific interactions contribute to increased network stability by preventing fluctuations in individual species from spreading throughout the entire network [14]. In the phyllosphere habitat, many associations within bacterial communities have been shown to be negative, primarily due to intense competition for limited and unevenly distributed resources and space [60]. In contrast, other studies indicate that facilitative interactions increase along a stress gradient, notably because stressful conditions may promote cooperation among species as they share resources or provide mutual support [21, 50]. Therefore, uncertainties remain regarding how needle aging influences the network stability of phyllosphere microbial communities.

In this study, our main objective was to investigate the variations in assembly processes, community composition and network properties of phyllosphere microbial communities across needle age cohorts. To do so, we collected 252 needle samples corresponding to three age cohorts (i.e., ‘young’, ‘mature’, and ‘old’) from three representative evergreen coniferous species (i.e., *Pinus koraiensis*, *Picea koraiensis*, and *Abies nephrolepis*) in temperate forests. We examined needle traits and determined both phyllosphere epiphytic bacterial and fungal communities using amplicon sequencing. We hypothesized that (H_1_) changes in needle traits (e.g., decreasing nutrient contents or increasing secondary metabolites) would increase deterministic selection with needle aging, thereby leading to greater similarity in microbial community composition [23]. Second, we hypothesized that (H_2_) microbial diversity would increase from young needles towards old needles due to the cumulative effects of neutral dispersal and random drift over time [44]. Third, we hypothesized that (H_3_) increasing microbial richness would in turn enhance network complexity (i.e., more species and interspecific associations), notably because it can create more opportunities for microbial taxa to interact with each other [71]. Finally, we expected that (H_4_) increasing microbial network complexity would lead to higher network stability (estimated by modularity and cohesion) in old needles compared with young needles, although previous studies indicated that network stability depended not only on the interaction intensity but also on the interaction direction (i.e., competition or cooperation) [14]. This is because complex interactions facilitate sustained resource exchange among community members, providing buffering effects to environmental stress and increasing community resistance [84].

## Materials and methods

### Site description

This research was conducted in natural mixed broadleaved-Korean pine forests in Shengshan (SS), Fenglin (FL), Liangshui (LS), Muling (ML), and Changbaishan (CBS) across Northeast China (126.78°-128.12°E, 42.32°-49.48°N) (Fig. S1). This region is recognized as one of the three major temperate mixed forest zones in the world [75]. *Pinus koraiensis*, *Picea koraiensis*, and *Abies nephrolepis* are the dominant evergreen coniferous species, and *Betula platyphylla*, *Acer pictum*, *Fraxinus mandshurica*, and *Quercus mongolica* are the dominant broadleaved species [41]. Specifically, *Abies* was missing in Shengshan (SS).

### Sample collection and chemical analysis

In August 2023, considering that the phyllosphere communities experience greater fluctuations in nutrients and climate conditions compared with soil communities [83], we collected needle samples from 6 individuals per tree species at each sampling site, with more than 10 m separating any two individuals [46, 72]. In order to obtain different needle age cohorts while ensuring enough needle quantity, needles were categorized into three age cohorts based on polycyclic shoots [17]: ‘young’ needles from 1-year-old branches, ‘mature’ needles from 3-year-old branches, and ‘old’ needles from 5-year-old or older branches. Consequently, any potential spatial autocorrelation among tree individuals would be evenly distributed across all three needle age cohorts, minimizing the impacts of spatial pseudoreplication on the main effect (i.e., needle age). Approximately 50 g of healthy needles per age cohort were randomly collected from four branches extending toward the east, south, west, and north in the subcanopy (about 8–12 m above the ground) of each individual. A total of 252 needle samples were collected in this study (5 sampling sites × 3 tree species × 6 individuals × 3 needle age cohorts). Subsequently, needle samples were randomly divided into two subsamples with equal mass for measuring needle traits and separating phyllosphere microorganisms, respectively.

Needles were oven-dried and then ground with a ball mill. Total carbon (TC) was determined by an automatic elemental analyzer (multi N/C 2100 S, analytikjena, Germany). Total nitrogen (TN) and total phosphorus (TP) were estimated by a laser-induced breakdown spectroscopy system (J200 LIBS; Applied Spectra, USA) [47]. Moreover, given the profound impacts of specialized host-derived secondary metabolites, such as flavonoids and tannins, on plant-microbe interactions [22, 73], we subsequently determined these two compounds with NaNO_2_-Al(NO_3_)_3_-NaOH complexometry and Folin-Ciocalteau reagent, respectively [32, 49]. More details are provided in Appendix S1 in Supporting Information.

### Phyllosphere microbial DNA extraction and sequencing

Phyllosphere epiphytic microorganisms were isolated by ultrasonication of 24 g needles in 240 mL sterilized ultra-pure water for 1 min, vortexing for 10 s, and then repeating these procedures once [9]. The suspension was filtered through a 0.22 μm filter, and which was then used for extracting microbial DNA with FastDNA™ Spin Kit for Soil (MP Biomedicals, USA) according to the manufacturer’s protocol. Primer pair 515 F (5’-GTGCCAGCMGCCGCGG-3’) − 907R (5’- CCGTCAATTCMTTTRAGTTT-3’) was used to amplify the V4-V5 region of bacterial 16 S rRNA [82], and ITS3F (5’-GCATCGATGAAGAACGCAGC-3’) - ITS4R (5’-TCCTCCGCTTATTGATATGC-3’) was used to amplify the fungal ITS2 region [65]. The amplification conditions were: initial denaturation at 95 °C for 3 min, 27 cycles of denaturation at 95 ℃ for 30 s, annealing at 55 ℃ for 30 s and extension at 72 ℃ for 45 s with a final extension at 72 ℃ for 10 min for bacteria and 35 cycles for fungi. The triplicate PCR products for each sample were pooled, purified using an AxyPrep DNA Gel Extraction Kit (Axygen, USA), and then quantified using a Qubit 4.0 spectrophotometer (Thermo Fisher Scientific, USA). Negative controls without template were included to evaluate the potential contamination during amplification.

PCR products were sequenced using the Illumina MiSeq PE300 platform. Sequences were quality trimmed with fastp v0.19.6 (low-quality reads with length < 50 bp or with a quality value < 20 or having N bases were discarded), merged using FLASH v1.2.11, and then de-noised with DADA2 in Qiime2 v2020.2 to obtain amplicon sequence variants (ASVs) [10]. After filtering out the sequences aligned to chloroplast and mitochondrial sequences, we rarefied the sequences of each sample to a minimum number of sequences (6730 for bacteria and 26604 for fungi) to reduce the interference of sequencing depth (Fig. S2). We finally obtained a total of 60,770 ASVs for bacteria and 39,849 ASVs for fungi across all 252 needle samples. Bacterial and fungal taxonomic assignments were performed using the Naive Bayes classifier in Qiime2 against the Silva 138 database and the Unite 8.0 database, respectively. We classified fungal ASVs into functional guilds based on the FUNGuild algorithm at the ‘Highly Probable’ and ‘Probable’ confidence rankings [54].

### Microbial network complexity analysis

To assess the potential interactions within both phyllosphere bacterial and fungal communities, SparCC networks were constructed for each needle age cohort with the *SpiecEasi* package [35]. SparCC is an algorithm considering sparse compositional data in network construction, and shows more robustness than the traditional correlation analysis [20]. Particularly, ASVs appearing in more than one-third of all samples were retained to diminish the impact of rare taxa in order to enhance the network reliability [61]. We used 100 permutations to calculate the significance of pairwise correlations of all ASVs, and then retained correlation matrices with correlation > 0.4 and significance < 0.05 [77]. Subsequently, multiple topological features were calculated. A network is considered to be more complex if it exhibits increased nodes (species), edges (associations among species), degree (average number of associations per species), clustering coefficient (how well a species is connected with its neighbors, and if a species is fully connected to its neighbors, the clustering coefficient is 1), and graph density (ratio of total associations to possible associations), alongside decreased average path length (average shortest distance between all pairs of species) [16]. We then calculated a network complexity index by averaging these topological features after they were standardized (ranging from 0 to 1) [30].

### Microbial network stability analysis

Network stability was evaluated with modularity and cohesion [26, 81]. A module is defined as a highly connected cluster of species that has fewer connections with other clusters. Module reflects ecological niche partitioning, and species in the same module usually have similar habitat preferences and functional associations [86]. We generated network modules using the greedy modularity optimization approach [53]. Higher modularity (i.e., close to 1) reflects that more connections appear within modules rather than among modules, and is desirable for the stability of biological networks. This is because the impacts of environmental disturbance on species will be limited to their own module and will not spread [57].

Cohesion is an abundance-weighted, null model-corrected metric based on the pairwise correlations of all species, which quantifies the connectivity of a microbial community (Eqs. 1–2) [27]:

1 $$\:{Cohesion}_{j}^{pos}=\:\sum\:_{i=1}^{n}{a}_{i}\times\:{c}_{i,\:i\:>\:0}$$

2 $$\:{Cohesion}_{j}^{neg}=\:\sum\:_{i=1}^{n}{a}_{i}\times\:{c}_{i,\:i<\:0}$$

where $\:{a}_{i}$ is the abundance of species *i* in sample *j*. *n* is the total number of species. $\:{c}_{i,\:i\:>\:0}$ and $\:{c}_{i,\:i<\:0}$ represent positive and negative connectedness, respectively. Positive cohesion and negative cohesion are bounded by 0 to 1 and − 1 to 0, respectively. Positive cohesion reflects the degree of cooperative behavior among community members, whereas negative cohesion indicates the magnitude of competitive behavior, and higher absolute values denote stronger interactions. We further defined the network cohesion as $\:Cohesion=|\:{Cohesion}_{j}^{neg}\:/\:{Cohesion}_{j}^{pos}\:|$, Microbial communities with lower negative cohesion but higher positive cohesion values are usually less stable, notably because positive interactions can lead to positive feedback loops, in which the disappearance of some species will cause the decline of other associated species [14].

### Statistical analysis

Richness was calculated to assess the α-diversity of both bacterial and fungal communities. To account for the compositional nature of the microbiome data, we applied centered log-ratio (CLR) transformation to the ASVs matrix using the *compositions* package [66], and β-diversity was estimated based on the Aitchison distance, and then visualized using principal coordinate analysis (PCoA). Permutational multivariate analysis of variance (PERMANOVA) was performed to test the effects of needle age on community composition using the ‘adonis2’ function in the *vegan* package [56]. Specifically, we defined the nested structure using the ‘blocks’ function by setting the interaction between sampling site, tree species, and individual identity as the block. The relative importance of stochastic and deterministic processes to community assembly was assessed according to Stegen et al., [63] using the *icamp* package [55]. Briefly, β-nearest taxon index (βNTI) > 2 indicates that the heterogeneous selection dominates the community, while βNTI < -2 represents homogeneous selection; |βNTI| < 2 with Bray-Curtis-based Raup-Crick (RC_bray_) > 0.95 or < -0.95 indicates the dominant effects of dispersal limitation or homogeneous dispersal, respectively, and |RC_bray_| < 0.95 represents ecological drift. Partial mantel tests were used to explore the major factors potentially influencing the assembly processes of phyllosphere microbial communities by comparing βNTI values with the Euclidean distance matrices of each needle trait after controlling for other traits using the ‘mantel.partial’ function in the *vegan* package. Redundancy analysis (RDA) was performed in Canoco 5.0 to determine the relative contribution of needle traits to network stability indices after eliminating collinearity (i.e., keeping variables with a variance inflation factor, VIF < 10).

Considering that we collected needle samples from more than one tree species per site, linear mixed effects models were applied to test how needle age (as a fixed factor) affected the richness, relative abundance, network complexity (including multiple individual variables and a comprehensive index), and network stability (i.e., modularity and cohesion) of both phyllosphere bacterial and fungal communities using the *lme4* package [7]. Sampling site, tree species, and individual identity were treated as random factors. Regarding model assumptions, residual normality was tested with the Shapiro-Wilk test, and homoscedasticity was assessed with residual plots. Specifically, data were Box-Cox transformed (i.e., the network modularity and cohesion of bacterial communities and the network complexity of fungal communities) with the *car* package to improve the residual normality [68]. Least significant difference (LSD) post-hoc test was conducted using the *emmeans* and *multcomp* packages if statistically significant effects of needle age (*p* < 0.05) were detected [28, 40].

## Results

### Microbial community composition was influenced by needle aging

**Fig. 1:**
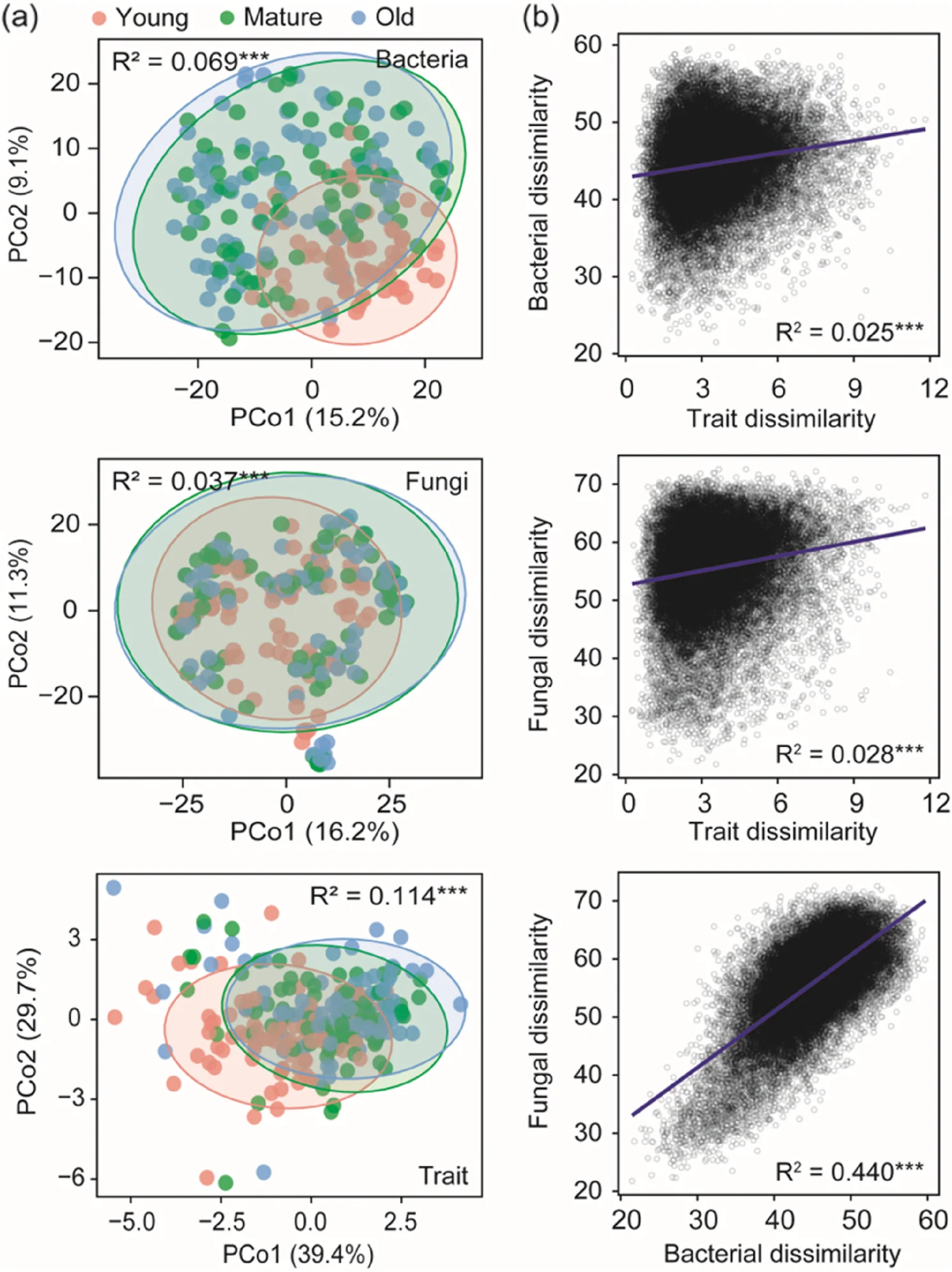
Principal coordinate analysis (PCoA) based on the composition of bacterial and fungal communities, as well as needle trait profile, across needle age cohorts (**a**). Relationships among the dissimilarity of bacterial communities, fungal communities, and needle trait profile (**b**). ***p < 0.001

PCoA ordinations and Adonis tests indicated that the composition of both phyllosphere bacterial and fungal communities, as well as needle trait profile, significantly varied during needle aging (*p* < 0.001) (Fig. 1a). Particularly, we observed that the relative abundance of Acidobacteriota, Actinomycetota, Bacteroidota, Myxococcota, Planctomycetota, saprophytic fungi, and symbiotic fungi increased over time (*p* < 0.05), while Gammaproteobacteria, Basidiomycota, and pathogenic fungi decreased (*p* < 0.05) (Fig. S3, Fig. S4). For needle traits, TP was the highest in young needles, followed by mature and old needles (*p* < 0.05), while TC: TP, TN: TP, tannins, and flavonoids increased from young needles towards old needles (*p* < 0.05) (Table S1). Overall, the compositional dissimilarity between mature and old needles was not significant for bacterial communities, fungal communities, and needle trait profile (*p* > 0.05), whereas significant dissimilarity was observed between young and mature needles, as well as between young and old needles (*p* < 0.001) (Table 1). There were positive correlations among the dissimilarity in composition of bacterial communities, fungal communities, and needle trait profile (*p* < 0.001) (Fig. 1b).

**Table 1:** Pairwise multilevel comparison of phyllosphere bacterial and fungal community composition and needle trait profile across needle age cohorts based on the permutational multivariate analysis of variance (PERMANOVA)

	Young vs. Mature		Young vs. Old		Mature vs. Old	
	*R* ^2^	*p*	*R* ^2^	*p*	*R* ^2^	*p*
Bacteria	0.066	< 0.001	0.090	< 0.001	0.007	0.212
Fungi	0.031	< 0.001	0.049	< 0.001	0.006	0.434
Trait	0.109	< 0.001	0.142	< 0.001	0.009	0.196

### Microbial community assembly changed across needle age cohorts

**Fig. 2:**
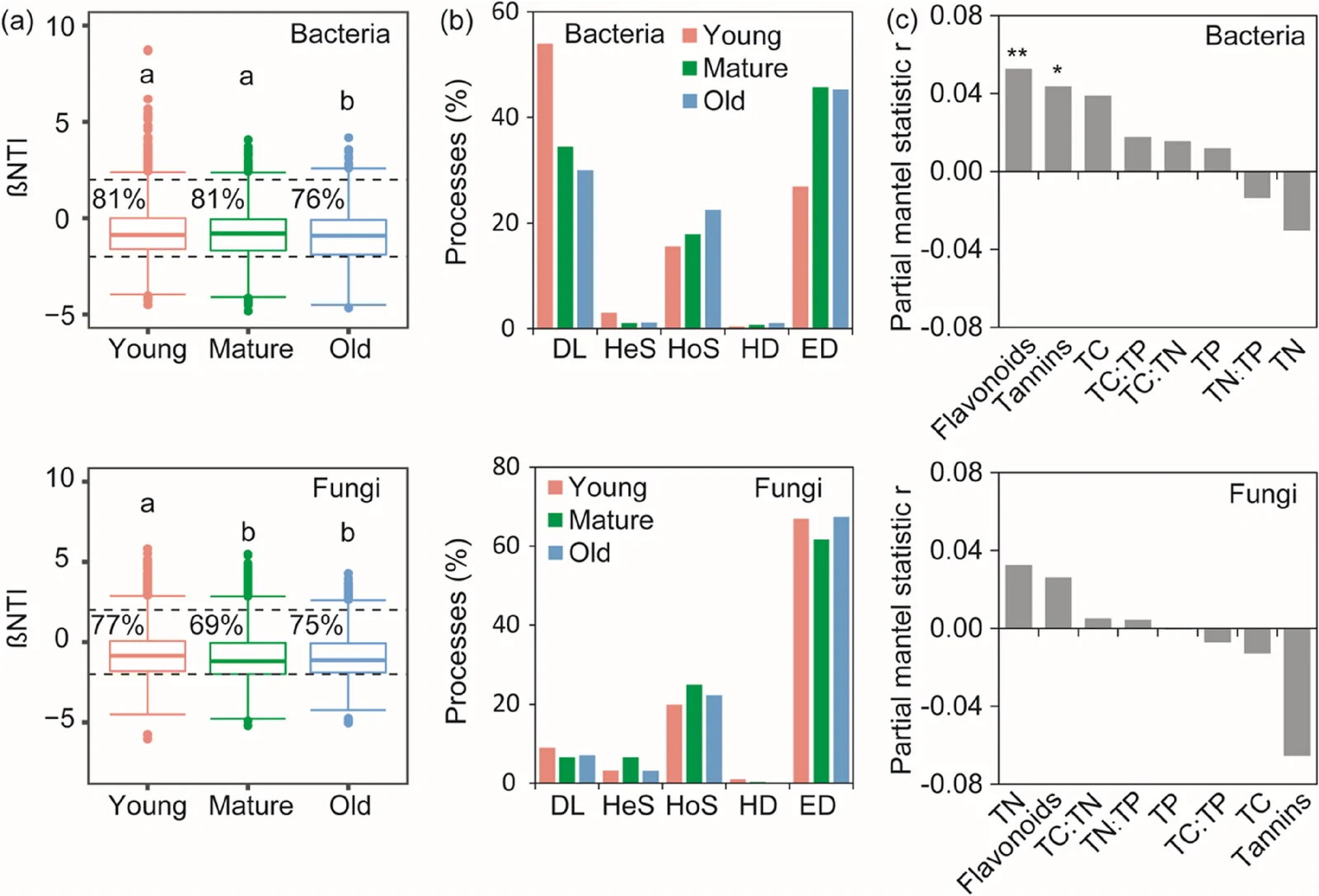
β-nearest taxon index (βNTI) of bacterial and fungal communities (**a**). Relative contributions of deterministic and stochastic processes to bacterial and fungal communities (**b**). DL, dispersal limitation; HeS, heterogeneous selection; HoS, homogeneous selection; HD, homogenizing dispersal; ED, ecological drift. Partial mantel tests of each needle trait against the βNTI of bacterial and fungal communities after controlling for other traits (**c**). TC, total carbon; TN, total nitrogen; TP, total phosphorus. Different lowercase letters indicate significant differences at *p* < 0.05. *p < 0.05 and **p < 0.01

When investigating the community assembly, we found that stochastic assembly governed both phyllosphere bacterial and fungal communities (> 60%), but its contribution decreased from young needles towards old needles (*p* < 0.001) (Fig. 2a, Fig. S5a). Specifically, dispersal limitation (DL) (> 10%) and ecological drift (ED) (> 24%) were more important than other processes for bacterial communities, with DL dominating young needles (> 30%) and ED dominating mature and old needles (> 36%) (Fig. 2b, Fig. S5b). Regarding fungal communities, ED had the strongest impact (> 51%) (Fig. 2b, Fig. S5b). Homogeneous selection (HoS) governed both bacterial (> 12%) and fungal (> 18%) communities, and increased during needle aging (Fig. 2b, Fig. S5b). Partial mantel tests unveiled that flavonoids were the key needle trait potentially affecting the assembly processes of both bacterial and fungal communities (Fig. 2c). Tannins enforced stronger impacts on bacterial communities compared with fungal communities, while fungal communities were influenced more by TN (Fig. 2c).

### Microbial diversity and network complexity increased during needle aging

**Fig. 3:**
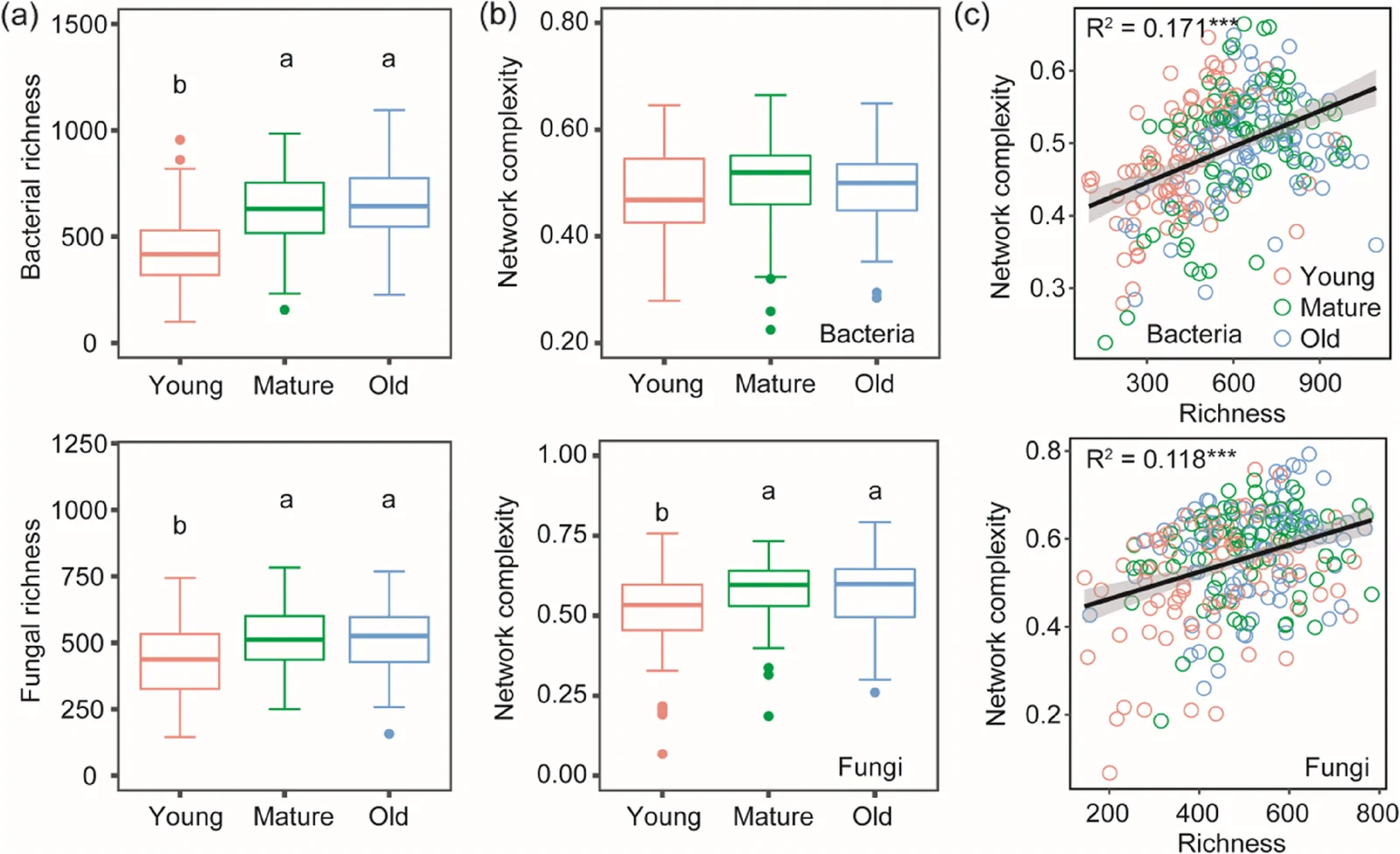
Richness (**a**) and network complexity (**b**) of bacterial and fungal communities. Correlations between richness and network complexity of bacterial and fungal communities (**c**). Different lowercase letters indicate significant differences at *p* < 0.05 when accounting for the random effects of tree species, sampling site, and individual identity. ***p < 0.001

The richness of both bacterial and fungal communities was higher in mature and old needles compared with young needles (*p* < 0.001) (Fig. 3a). Regarding network complexity features, we found that the node number, edge number, and degree of both bacterial and fungal communities, as well as the corrected average path length of fungal communities, increased in mature and old needles (*p* < 0.05), while the graph density and clustering coefficient of bacterial communities decreased (*p* < 0.05) (Fig. S6). Overall, the network complexity of bacterial communities did not change during needle aging (*p* > 0.05), whereas that of fungal communities increased (*p* < 0.001) (Fig. 3b). There were positive relationships between the richness and network complexity of both bacterial and fungal communities (*p* < 0.001) (Fig. 3c).

### Needle aging destabilized microbial networks

**Fig. 4:**
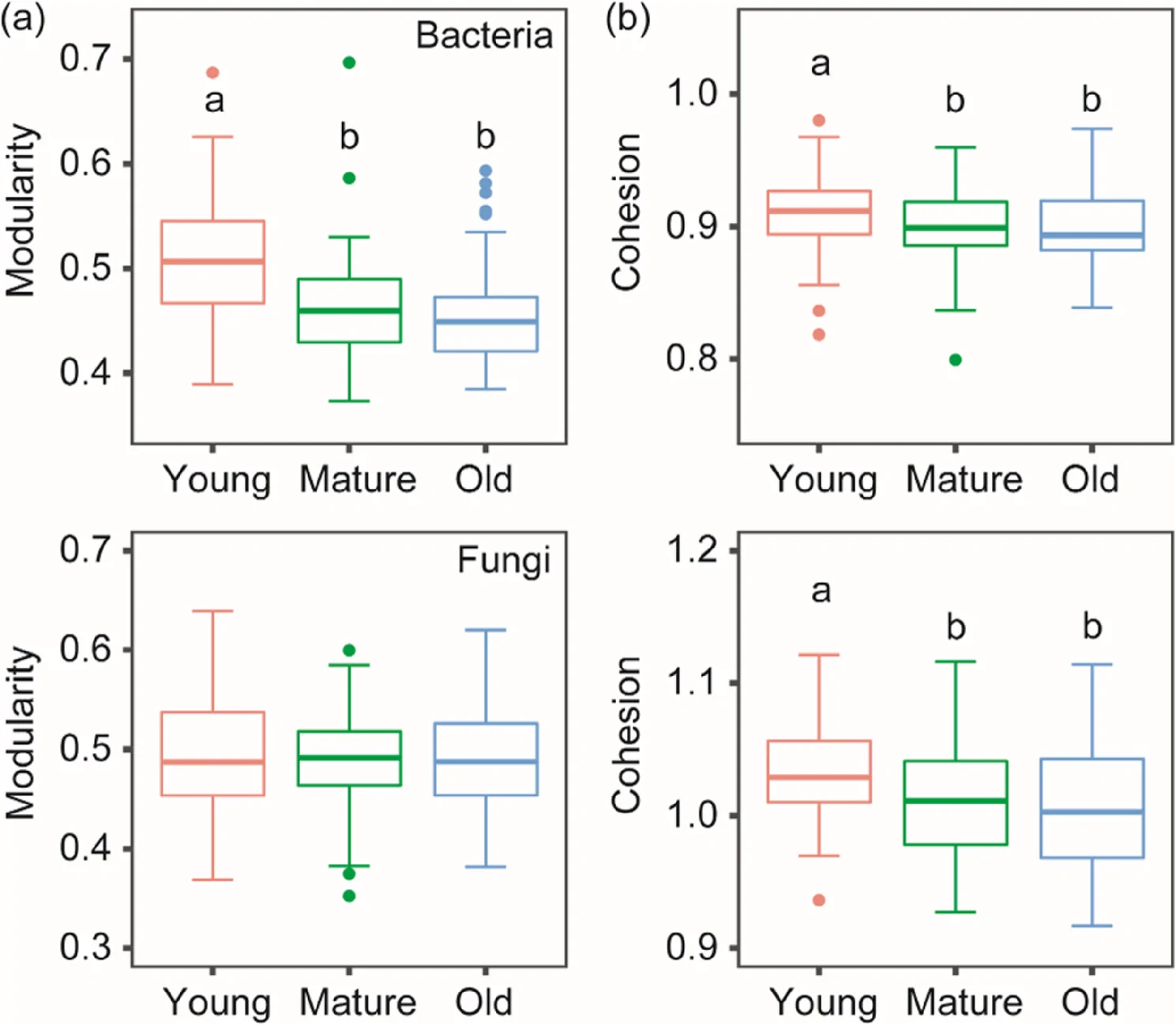
Modularity (**a**) and cohesion (**b**) used to describe the network stability of bacterial and fungal communities. Cohesion was calculated as the absolute value of negative cohesion to positive cohesion, representing the strength of negative species interactions to positive species interactions in networks. Different lowercase letters indicate significant differences at *p* < 0.05 when accounting for the random effects of tree species, sampling site, and individual identity

The network modularity of bacterial communities, but not fungal communities, varied across needle age cohorts, and decreased from young needles towards old needles (*p* < 0.05) (Fig. 4a). Moreover, the network cohesion (i.e., the ratio of negative cohesion to positive cohesion) of both bacterial and fungal communities was higher in young needles compared with mature and old needles (*p* < 0.05) (Fig. 4b). Specifically, the absolute values of negative cohesion (i.e., negative species interactions) and positive cohesion (i.e., positive species interactions) of bacterial networks gradually decreased during needle aging (*p* < 0.05) (Fig. S7a), whereas that of fungal networks increased (*p* < 0.05) (Fig. S7b). Redundancy analysis (RDA) indicated that the variations in the network stability of both bacterial and fungal communities during needle aging were closely correlated with the changes in flavonoids, followed by TP and tannins (*p* < 0.01) (Fig. S8a and b).

## Discussion

**Fig. 5:**
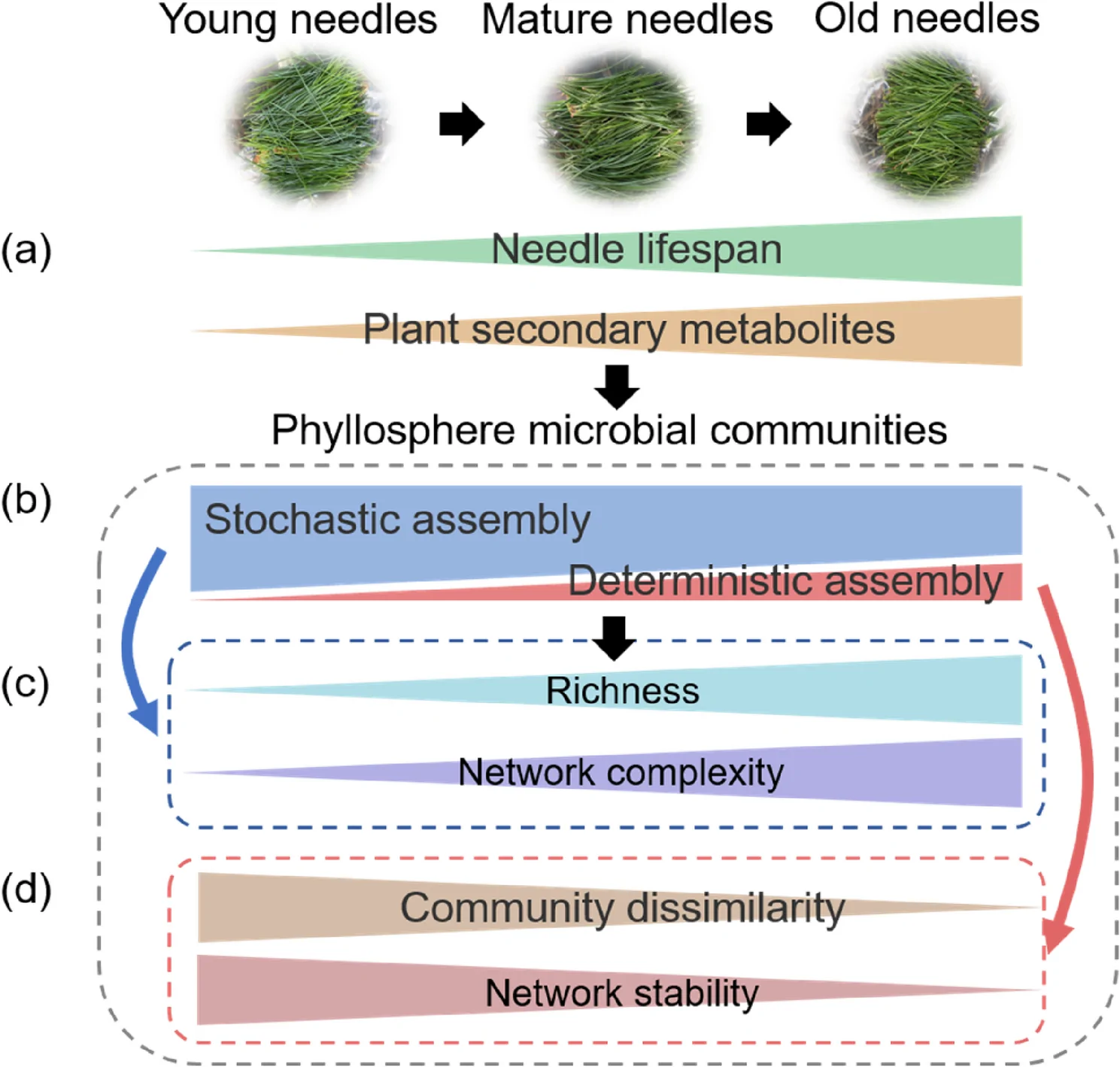
Conceptual model showing the succession of phyllosphere microbial communities across needle age cohorts. Needle lifespan and plant secondary metabolites increased during needle aging (**a**). Stochastic assembly dominated microbial communities, and decreased from young needles towards old needles, while deterministic assembly increased (**b**). Longer immigration periods increased microbial richness and network complexity (**c**). Stronger selective stress reduced community dissimilarity and network stability (**d**)

Our knowledge on diversity patterns of phyllosphere microbial communities has accumulated rapidly over the past few years [31, 37, 76]. However, few studies have investigated the assembly processes driving phyllosphere microbial communities across needle age cohorts. The present study showed that stochastic processes dominated both bacterial and fungal communities, leading to higher species diversity and network complexity in older needles. Increased deterministic processes with needle aging reduced community dissimilarity and network stability (Fig. 5).

### Needle aging generates decreased microbial community dissimilarity

In agreement with our first hypothesis (H_1_), we demonstrated that deterministic assembly increased during needle aging for both bacterial and fungal communities. Overall, we found that the dominance of copiotrophic bacteria (e.g., Gammaproteobacteria) in young needles was replaced by oligotrophic bacteria (e.g., Acidobacteriota) in mature and old needles. Moreover, we also found a decrease in pathogens and an increase in saprotrophs as well as symbiotrophs for fungi with needle aging. These changes were closely associated with the rising levels of phenolic compounds such as flavonoids, which can act as both antibacterial substances and signaling molecules involved in protecting plant to against the colonization of some microorganisms while recruiting other specific taxa [73]. This is in line with Carper et al. [11] who suggested that the decreased bacterial variations in older needles were related to the increased secondary metabolites potentially caused by historical herbivore attacks [13].

To a lesser extent, changes in nutrient content such as TN also contributed to shaping fungal community composition. This is because N is essential for amino acid synthesis, protein production, and hyphal growth [19]. These results are consistent with previous researches highlighting the combined role of leaf nutrients and secondary metabolites in regulating the structure and assembly of plant-associated microbiomes by modifying selection pressures [29, 43]. Although we should interpret these correlative results with caution, they seem to indicate that environmental conditions imposed by the host strengthen the homogenizing effects through enriching microbial taxa with strong adaptability, ultimately enhancing the community similarity over time [69]. Overall, these findings highlight the importance of needle traits in shaping the successions of phyllosphere microbial communities in evergreen coniferous species, as needle traits evolve across age cohorts.

### Microbial diversity increases with needle aging

In agreement with our second hypothesis (H_2_), we found that the diversity of both bacterial and fungal communities increased from young needles towards old needles. This is probably because the longer immigration time along the needle lifespan facilitates the accumulation and colonization of microorganisms from surrounding environmental sources through windborne dispersal, rain-assisted splashing, and insect activity [83]. In addition to the horizontal transmission, a part of phyllosphere epiphytic microorganisms can be vertically transferred from the interior of host plants (e.g., seeds) [1]. The longer-term co-evolution of these microbial taxa with host plants contributes to their coexistence in old needles [80]. In line with this idea, we found that the lower dispersal limitation, alongside higher ecological drift, occurred in old needles compared with young needles.

Moreover, needle aging also induces the shifts in microenvironment, which can control fine-scale variations in microbial colonization, survival, and ultimately diversity [25, 72]. UV radiation typically decreases from young needles towards old needles [12], leading to increased shaded conditions and air moisture, which may further facilitate the diversity of both bacterial and fungal communities [39]. This is consistent with recent studies showing that stochastic processes are powerful drivers of the microbial immigration, as they can introduce new species from regional pools that are not present in the initial community [33, 85]. As such, community assembly is shaped by the balance between stochastic processes (immigration and ecological drift) and deterministic processes (environmental filtering), with deterministic forces increasing over time, but stochastic processes remaining dominant throughout needle life stages.

### Increasing microbial diversity is accompanied by greater network complexity

In line with our third hypothesis (H_3_), we observed that network complexity increased from young needles towards old needles. Although it is typically believed that resource constraints will lead to simpler networks [42, 58], increasing microbial diversity may create more opportunities for microbial taxa to interact with each other, thereby supporting greater network complexity [71]. In addition, a longer coexistence period may enhance the relative fitness of microorganisms and their interactions, partially compensating for the adverse effects in the phyllosphere habitat [3, 51]. Furthermore, challenging resource conditions (e.g., secondary metabolites and nutrient limitation) in old needles may favor the presence of stress-tolerant taxa [26]. For instance, Wang et al. [72] found that Actinomycetota was dominant in perennial needles, and the majority of this phylotype can degrade complex organic materials, thus helping to enhance the community adaptability through establishing more links with other members [5]. Altogether, these results suggest that microbial communities become more connected during needle aging, and emphasize the dominant role of stochastic assembly in maintaining phyllosphere microbial diversity and interaction patterns.

### Needle aging reduced the network stability of microbial communities

Contrary to our last hypothesis (H_4_), we found that the network stability of both bacterial and fungal communities decreased during needle aging. These results challenge the traditional view that complexity confers stability [18, 52]. Instead, we highlighted that plant secondary metabolites may disrupt established microbial interactions by creating selective pressures, thereby destabilizing microbial networks, although closely connected networks typically offer resilience against environmental fluctuations [41]. A network module represents a group of microbial taxa that share similar niches or have a relatively similar phylogeny [74]. Higher environmental stress may promote the associations among microorganisms with different habitat preferences [26]. Here, we showed that microbial networks in old needles were less modular, indicating that more associations occurred among modules rather than within them [48]. This can lead to a widespread propagation of species fluctuations within one module to species in other modules [64].

Additionally, increasing selective stress with needle aging may promote mutualistic symbiosis by replacing competitive taxa that engage in antagonistic interactions with stress-tolerant and facilitative taxa [21, 50]. In this study, we observed a reduced ratio of negative cohesion to positive cohesion from young needles towards old needles for both bacterial and fungal communities. Together with this, pathogenic fungi decreased but symbiotic fungi increased in mature and old needles, leading to increased positive cohesion in the fungal communities. This supports the ecological mechanisms underlying the Stress Gradient Hypothesis that facilitative interactions may become more prevalent as environmental stress intensifies [8]. However, strong positive feedback loops among species will enhance their dependence on each other, leading to a rapid spread of perturbations from individuals to the entire system [14].

Our study provides novel insights into the succession and drivers of phyllosphere microbial communities during needle aging. Nevertheless, given the dispersal capacity of microorganisms and environmental gradients of forest ecosystems, the crown diameter or separation distance between tree individuals may influence the assembly processes of phyllosphere communities, and this should be carefully taken into account in the future.

## Conclusions

Our results offer comprehensive empirical evidence on the leading role of stochastic processes in phyllosphere microbial communities across needle age cohorts. We highlighted that longer immigration periods induced higher microbial diversity and network complexity in older needles, while lower community dissimilarity and network stability were closely related to higher concentrations of plant secondary metabolites and lower nutrient contents. These findings deepen our understanding of plant-microbiome interactions, and emphasize that the developmental stages of host plants is a key factor that should not be overlooked when explaining the variations in plant microbiomes. Moreover, the progressive evolution of needle traits such as the accumulation of secondary metabolites, rather than nutrient depletion alone, plays a critical role in shaping microbial communities, and this must be considered in future studies.

## Supplementary Information

Supplementary Material 1: Appendix S1. Needle trait dataset.

Supplementary Material 2: Appendix S2. Supplementary table and figures. Table S1 Needle traits across age cohorts. Fig. S1 Location of sampling sites. SS, Shengshan; FL, Fenglin; LS, Liangshui; ML, Muling; CBS, Changbaishan. Fig. S2 Rarefaction curves of bacterial (a) and fungal (b) communities across different samples. Fig. S3 Dominant bacterial phyla (relative abundance > 1%). Different lowercase letters indicate significant differences at *p* < 0.05 when accounting for the random effects of tree species, sampling site, and individual identity. Fig. S4 Dominant fungal phyla (relative abundance > 1%) and functional guilds. Different lowercase letters indicate significant differences at *p* < 0.05 when accounting for the random effects of tree species, sampling site, and individual identity. Fig. S5 β-nearest taxon index (βNTI) of bacterial and fungal communities across different tree species (a). Relative contributions of deterministic and stochastic processes to bacterial and fungal communities (b). DL, dispersal limitation; HeS, heterogeneous selection; HoS, homogeneous selection; HD, homogenizing dispersal; ED, ecological drift. Different lowercase letters indicate significant differences at *p* < 0.05. Fig. S6 Multiple network features used to describe the network complexity of bacterial (a) and fungal (b) communities. Different lowercase letters indicate significant differences at *p* < 0.05 when accounting for the random effects of tree species, sampling site, and individual identity. Fig. S7 Absolute values of negative cohesion (i.e., negative species interactions) and positive cohesion (i.e., positive species interactions) of bacterial (a) and fungal (b) networks. Different lowercase letters indicate significant differences at *p* < 0.05 when accounting for the random effects of tree species, sampling site, and individual identity. Fig. S8 The relative effects of needle traits on multiple network stability indices of bacterial (a) and fungal (b) communities. *p < 0.05 and **p < 0.01.

## Data Availability

Raw sequencing data reported in this study have been deposited in the Genome Sequence Archive (GSA) with accession numbers: CRA029486.

## References

[1] Abdelfattah A, Tack AJM, Lobato C, Wassermann B, Berg G. From seed to seed: the role of microbial inheritance in the assembly of the plant microbiome. Trends Microbiol. 2023;31:346–55. 10.1016/j.tim.2022.10.009. 36481186

[2] Albert LP, Wu J, Prohaska N, de Camargo PB, Huxman TE, Tribuzy ES, Ivanov VY, Oliveira RS, Garcia S, Smith MN, Junior O, Restrepo-Coupe RC, da Silva N, Stark R, Martins SC, Penha GA, D. V., Saleska SR. Age-dependent leaf physiology and consequences for crown-scale carbon uptake during the dry season in an Amazon evergreen forest. New Phytol. 2018;219:870–84. 10.1111/nph.15056. 29502356

[3] Allison SD, Martiny JBH. Resistance, resilience, and redundancy in microbial communities. Proc Natl Acad Sci USA. 2008;105:11512–9. 10.1073/pnas.0801925105. 18695234 PMC2556421

[4] Banerjee S, Walder F, Büchi L, Meyer M, Held AY, Gattinger A, Keller T, Charles R, van der Heijden MG A. Agricultural intensification reduces microbial network complexity and the abundance of keystone taxa in roots. ISME J. 2019;13:1722–36. 10.1038/s41396-019-0383-2. 30850707 PMC6591126

[5] Bao YY, Dolfing J, Guo ZY, Chen RR, Wu M, Li ZP, Lin XG, Feng YZ. Important ecophysiological roles of non-dominant Actinobacteria in plant residue decomposition, especially in less fertile soils. Microbiome. 2021;9:84. 10.1186/s40168-021-01032-x. 33827695 PMC8028251

[6] Barnes AD, Deslippe JR, Potapov AM, Romero-Olivares AL, Schipper LA, Alster CJ. Does warming erode network stability and ecosystem multifunctionality? Trends Ecol Evol. 2024;39:892–4. 10.1016/j.tree.2024.08.006. 39217061

[7] Bates DM, Mächler M, Bolker BM, Walker S. Fitting linear mixed-effects models using lme4. J Stat Softw. 2015;67:1–48. 10.18637/jss.v067.i01.

[8] Bertness MD, Callaway R. Positive interactions in communities. Trends Ecol Evol. 1994;9:191–3. 10.1016/0169-5347(94)90088-4. 21236818

[9] Bodenhausen N, Horton MW, Bergelson J. Bacterial communities associated with the leaves and the roots of *Arabidopsis thaliana*. PLoS ONE. 2013;8:e56329. 10.1371/journal.pone.0056329. 23457551 PMC3574144

[10] Callahan BJ, McMurdie PJ, Rosen MJ, Han AW, Johnson AJA, Holmes SP. DADA2: High-resolution sample inference from Illumina amplicon data. Nat Methods. 2016;13:581–3. 10.1038/NMETH.3869. 27214047 PMC4927377

[11] Carper DL, Lawrence TJ, Quiroz D, Kueppers LM, Frank C, A. Needle bacterial community structure across the species range of limber pine. ISME Commun. 2024;4:ycae062. 10.1093/ismeco/ycae062. 38800125 PMC11128189

[12] Cescatti A, Niinemets Ü. Sunlight capture. Leaf to landscape. In: Smith WK, Vogelmann TC, Chritchley C, editors. Photosynthetic adaptation. Chloroplast to landscape. Berlin: Springer; 2004. pp. 42–85.

[13] Clark EL, Carroll AL, Huber DPW. Differences in the constitutive terpene profile of lodgepole pine across a geographical range in British Columbia, and correlation with historical attack by mountain pine beetle. Can Entomol. 2010;142:557–73. 10.4039/n10-022.

[14] Coyte KZ, Schluter J, Foster KR. The ecology of the microbiome: networks, competition, and stability. Science. 2015;350:663–6. 10.1126/science.aad2602. 26542567

[15] de Vries FT, Griffiths RI, Bailey M, Craig H, Girlanda M, Gweon HS, Hallin S, Kaisermann A, Keith AM, Kretzschmar M, Lemanceau P, Lumini E, Mason KE, Oliver A, Ostle N, Prosser JI, Thion C, Thomson B, Bardgett RD. Soil bacterial networks are less stable under drought than fungal networks. Nat Commun. 2018;9:3033. 10.1038/s41467-018-05516-7. 30072764 PMC6072794

[16] Deng Y, Jiang YH, Yang YF, He ZL, Luo F, Zhou JZ. Molecular ecological network analyses. BMC Bioinformatics. 2012;13:113. 10.1186/1471-2105-13-113. 22646978 PMC3428680

[17] Eimil-Fraga C, Sanchez-Rodriguez F, Alvarez-Rodriguez E, Rodriguez-Soalleiro R. Relationships between needle traits, needle age and site and stand parameters in *Pinus pinaster*. Trees-Structure Function. 2015;29:1103–13. 10.1007/s00468-015-1190-7.

[18] Fan KK, Weisenhorn P, Gilbert JA, Chu HY. Wheat rhizosphere harbors a less complex and more stable microbial co-occurrence pattern than bulk soil. Soil Biol Biochem. 2018;125:251–60. 10.1016/j.soilbio.2018.07.022.

[19] Fanin N, Hättenschwiler S, Soria PFC, Fromin N. (A)synchronous availabilities of N and P regulate the activity and structure of the microbial decomposer community. Front Microbiol. 2016;6:1507. 10.3389/fmicb.2015.01507. 26779162 PMC4701990

[20] Friedman J, Alm EJ. Inferring Correlation Networks from genomic survey data. PLoS Comput Biol. 2012;8:e1002687. 10.1371/journal.pcbi.1002687. 23028285 PMC3447976

[21] Garcia MO, Templer PH, Sorensen PO, Sanders-DeMott R, Groffman PM, Bhatnagar JM. Soil microbes trade-off biogeochemical cycling for stress tolerance traits in response to year-round climate change. Front Microbiol. 2020;11:616. 10.3389/fmicb.2020.00616. 32477275 PMC7238748

[22] Haase K, Wantzen KM. Analysis and decomposition of condensed tannins in tree leaves. Environ Chem Lett. 2008;6:71–5. 10.1007/s10311-008-0140-7.

[23] Han X, Li YB, Li YH, Du XF, Li B, Li Q, Bezemer TM. Soil inoculum identity and rate jointly steer microbiomes and plant communities in the field. ISME Commun. 2022;2:59. 10.1038/s43705-022-00144-1. 37938291 PMC9723724

[24] Hassani MA, Durán P, Hacquard S. Microbial interactions within the plant holobiont. Microbiome. 2018;6(58). 10.1186/s40168-018-0445-0. PMC5870681 29587885

[25] Heil JA, Simler-Williamson A, Striluk ML, Trawick D, Capezza R, Defehr C, Osorio A, Finney B, Turner KG, Bittleston LS. Weather and leaf age separately contribute to temporal shifts in phyllosphere fungal community structure in sagebrush. Ecosphere. 2025;16:6. 10.1002/ecs2.70295.

[26] Hernandez DJ, David AS, Menges ES, Searcy CA, Afkhami ME. Environmental stress destabilizes microbial networks. ISME J. 2021;15:1722–34. 10.1038/s41396-020-00882-x. 33452480 PMC8163744

[27] Herren CM, McMahon KD. Cohesion: a method for quantifying the connectivity of microbial communities. ISME J. 2017;11:2426–38. 10.1038/ismej.2017.91. 28731477 PMC5649174

[28] Hothorn T, Bretz F, Westfall P, Heiberger RM, Schuetzenmeister A, Scheibe S. multcomp: simultaneous inference in general parametric models. R package version. 2021;1:4–17. https://CRAN.R-project.org/package=multcomp.

[29] Ivey KL, Chan AT, Izard J, Cassidy A, Rogers GB, Rimm EB. Role of dietary flavonoid compounds in driving patterns of microbial community assembly. mBio. 2019;10:e01205–19. 10.1128/mBio.01205-19. 31551328 PMC6759757

[30] Jiao S, Qi JJ, Jin CJ, Liu Y, Wang Y, Pan HB, Chen S, Liang CL, Peng ZH, Chen BB. Core phylotypes enhance the resistance of soil microbiome to environmental changes to maintain multifunctionality in agricultural ecosystems. Glob Change Biol. 2022;28:6653–64. 10.1111/gcb.16387. 36002985

[31] Kembel SW, O’Connor TK, Arnold HK, Hubbell SP, Wright SJ, Green JL. Relationships between phyllosphere bacterial communities and plant functional traits in a neotropical forest. Proc Natl Acad Sci USA. 2014;111:13715–20. 10.1073/pnas.1216057111. 25225376 PMC4183302

[32] Kim DO, Jeong SW, Lee CY. Antioxidant capacity of phenolic phytochemicals from various cultivars of plums. Food Chem. 2003;81:321–6. 10.1016/S0308-8146(02)00423-5.

[33] Knelman JE, Nemergut DR. Changes in community assembly may shift the relationship between biodiversity and ecosystem function. Front Microbiol. 2014;5:424. 10.3389/fmicb.2014.00424. 25165465 PMC4131499

[34] Koskella B. The phyllosphere. Curr Biol. 2020;30:R1096–169. 10.1016/j.cub.2020.07.037. 33022257

[35] Kurtz Z, Mueller C, Miraldi E, Bonneau R, Tipton L. (2021). SpiecEasi: Sparse inverse covariance for ecological statistical inference. R package version 1.1.0. https://CRAN.R-project.org/package=SpiecEasi.

[36] Kuusk V, Niinemets Ü, Valladares F. A major trade-off between structural and photosynthetic investments operative across plant and needle ages in three Mediterranean pines. Tree Physiol. 2018;38:543–57. 10.1093/treephys/tpx139. 29281105

[37] Laforest-Lapointe I, Messier C, Kembel SW. Host species identity, site and time drive temperate tree phyllosphere bacterial community structure. Microbiome. 2016;4:27. 10.1186/s40168-016-0174-1. 27316353 PMC4912770

[38] Laforest-Lapointe I, Paquette A, Messier C, Kembel SW. Leaf bacterial diversity mediates plant diversity and ecosystem function relationships. Nature. 2017;546:145–7. 10.1038/nature22399. 28538736

[39] Lei CT, Zhou SYD, Tissue DT, Neilson R, Lie ZY, Wu T, Liu XJ, Meng CS, Li X, Zhu D, Liu JX. Seasonal variation of phyllosphere microbial communities under warming. Glob Change Biol. 2025;31:e70270. 10.1111/gcb.70270. 40452412

[40] Lenth RV, Buerkner P, Herve M, Love J, Riebl H, Singmann H. (2021). emmeans: estimated marginal means, aka least-squares means. R package version 1.6.0. https://CRAN.R-project.org/package=emmeans.

[41] Li B, Fu MY, Jin GZ, Liu ZL. Rare bacterial subcommunity drives nutrient cycling in phyllosphere habitat of evergreen conifers. Microbiol Spectr. 2025;13:8. 10.1128/spectrum.00518-25. PMC12323584 40662582

[42] Li B, Li YB, Fanin N, Han X, Du XF, Liu HW, Li YH, Li Q. Adaptation of soil micro-food web to elemental limitation: evidence from the forest-steppe ecotone. Soil Biol Biochem. 2022a;170:108698. 10.1016/j.soilbio.2022.108698.

[43] Li MJ, Hong L, Ye WH, Wang ZM, Shen H. Phyllosphere bacterial and fungal communities vary with host species identity, plant traits and seasonality in a subtropical forest. Environ Microbiome. 2022b;17:29. 10.1186/s40793-022-00423-3. 35681245 PMC9185928

[44] Liang YT, Ning DL, Lu ZM, Zhang N, Hale L, Wu LY, Clark IM, McGrath SP, Storkey J, Hirsch PR, Sun B, Zhou JZ. Century long fertilization reduces stochasticity controlling grassland. Soil Biol Biochem. 2020;151:108023. 10.1016/j.soilbio.2020.108023.

[45] Liu C, Jin GZ, Liu ZL. Importance of organ age in driving intraspecific trait variation and coordination for three evergreen coniferous species. Ecol Ind. 2021;121:107099. 10.1016/j.ecolind.2020.107099.

[46] Liu ZL, Hikosaka K, Li FR, Jin GZ. Variations in leaf economics spectrum traits for an evergreen coniferous species: tree size dominates over environment factors. Funct Ecol. 2020;34:458–67. 10.1111/1365-2435.13498.

[47] Lu CP, Wang LS, Hu HY, Zhuang Z, Wang YB, Wang RJ, Song LT. Analysis of total nitrogen and total phosphorus in soil using laser-induced breakdown spectroscopy. Chin Opt Lett. 2013;11:053004. 10.3788/COL201311.053004.

[48] Luo S, Png GK, Ostle NJ, Zhou HK, Hou XY, Luo CL, Quinton JN, Schaffner U, Sweeney C, Wang DJ, Wu JH, Wu YW, Bardgett RD. Grassland degradation-induced declines in soil fungal complexity reduce fungal community stability and ecosystem multifunctionality. Soil Biol Biochem. 2023;176:108865. 10.1016/j.soilbio.2022.108865.

[49] Makkar HPS. Quantification of tannins in tree and shrub foliage: a laboratory manual. Netherlands: Kluwer Academic; 2003.

[50] Männistö M, Ganzert L, Tiirola M, Häggblom MM, Stark S. Do shifts in life strategies explain microbial community responses to increasing nitrogen in tundra soil? Soil Biol Biochem. 2016;96:216–28. 10.1016/j.soilbio.2016.02.012.

[51] Morriën E, Hannula SE, Snoek LB, Helmsing NR, Zweers H, de Hollander M, Soto RL, Bouffaud ML, Buée M, Dimmers W, Duyts H, Geisen S, Girlanda M, Griffiths RI, Jorgensen HB, Jensen J, Plassart P, Redecker D, Schmelz RM, van der Putten WH. Soil networks become more connected and take up more carbon as nature restoration progresses. Nat Commun. 2017;8:14349. 10.1038/ncomms14349. 28176768 PMC5309817

[52] Mougi A, Kondoh M. Diversity of interaction types and ecological community stability. Science. 2012;337:349–51. 10.1126/science.1220529. 22822151

[53] Newman MEJ. Finding community structure in networks using the eigenvectors of matrices. Phys Rev E. 2006;74:036104. 10.1103/PhysRevE.74.036104. 17025705

[54] Nguyen NH, Song ZW, Bates ST, Branco S, Tedersoo L, Menke J, Schilling JS, Kennedy PG. FUNGuild: An open annotation tool for parsing fungal community datasets by ecological guild. Fungal Ecol. 2016;20:241–8. 10.1016/j.funeco.2015.06.006.

[55] Ning DL, Yuan MT, Wu LW, Zhang Y, Guo X, Zhou XS, Yang YF, Arkin AP, Firestone MK, Zhou JZ. A quantitative framework reveals ecological drivers of grassland microbial community assembly in response to warming. Nat Commun. 2020;11:4717. 10.1038/s41467-020-18560-z. 32948774 PMC7501310

[56] Oksanen J, Blanchet FG, Friendly M, Kindt R, Legendre P, McGlinn D, O’Hara RB, Simpson GL, Solymos P, Stevens MH, Szoecs E, Wagner H. (2020). vegan: community ecology package. R package version 2.5-7. https://CRAN.R-project.org/package=vegan.

[57] Olesen JM, Bascompte J, Dupont YL, Jordano P. The modularity of pollination networks. Proc Natl Acad Sci USA. 2007;104:19891–6. 10.1073/pnas.0706375104. 18056808 PMC2148393

[58] Qiu LP, Zhang Q, Zhu HS, Reich PB, Banerjee S, van der Heijden MGA, Sadowsky MJ, Ishii S, Jia XX, Shao MG, Liu BY, Jiao H, Li HQ, Wei XR. Erosion reduces soil microbial diversity, network complexity and multifunctionality. ISME J. 2021;15:2474–89. 10.1038/s41396-021-00913-1. 33712698 PMC8319411

[59] Santolini M, Barabási AL. (2018). Predicting perturbation patterns from the topology of biological networks. Proceedings of the National Academy of Sciences, USA, 115, E6375-E6383. 10.1073/pnas.1720589115. PMC6142275 29925605

[60] Schlechter RO, Kear EJ, Bernach M, Remus DM, Remus-Emsermann MNP. Metabolic resource overlap impacts competition among phyllosphere bacteria. ISME J. 2023;17:1445–54. 10.1038/s41396-023-01459-0. 37355740 PMC10432529

[61] Shi Y, Delgado-Baquerizo M, Li YT, Yang YF, Zhu YG, Peñuelas J, Chu HY. Abundance of kinless hubs within soil microbial networks are associated with high functional potential in agricultural ecosystems. Environ Int. 2020;142:105869. 10.1016/j.envint.2020.105869. 32593837

[62] Smets W, Chock MK, Walsh CM, Vanderburgh CQ, Kau E, Lindow SE, Fierer N, Koskella B. Leaf side determines the relative importance of dispersal versus host filtering in the phyllosphere microbiome. mBio. 2023;14:01111–23. 10.1128/mbio.01111-23. PMC10470611 37436063

[63] Stegen JC, Lin XJ, Fredrickson JK, Chen XY, Kennedy DW, Murray CJ, Rockhold ML, Konopka A. Quantifying community assembly processes and identifying features that impose them. ISME J. 2013;7:2069–79. 10.1038/ismej.2013.93. 23739053 PMC3806266

[64] B Stouffer D, Bascompte J. Compartmentalization increases food-web persistence. Proc Natl Acad Sci USA. 2011;108:3648–52. 10.1073/pnas.1014353108. 21307311 PMC3048152

[65] Toju H, Tanabe AS, Yamamoto S, Sato H. High-coverage ITS primers for the DNA-based identification of ascomycetes and basidiomycetes in environmental samples. PLoS ONE. 2012;7:e40863. 10.1371/journal.pone.0040863. 22808280 PMC3395698

[66] van den Boogaart KG, Tolosana-Delgado R, Bren M. (2025). Compositions: compositional data analysis. R package version 2.0–9. https://CRAN.R-project.org/package=Compositions.

[67] van der Wal A, Leveau JHJ. Modelling sugar diffusion across plant leaf cuticles: the effect of free water on substrate availability to phyllosphere bacteria. Environ Microbiol. 2011;13:792–7. 10.1111/j.1462-2920.2010.02382.x. 21091864

[68] Velilla S. A note on the multivariate Box-Cox transformation to normality. Stat Probab Lett. 1993;17:259–63. 10.1016/0167-7152(93)90200-3.

[69] Viviane C, Francisco DA, Víctor JC, Jos MR. Ecology and evolution of plant microbiomes. Annual Rev Microbiol. 2019;73:69–88. 10.1146/annurev-micro-090817-062524. 31091418

[70] Vorholt JA. Microbial life in the phyllosphere. Nat Rev Microbiol. 2012;10:828–40. 10.1038/nrmicro2910. 23154261

[71] Wagg C, Schlaeppi K, Banerjee S, Kuramae EE, van der Heijden MGA. Fungal-bacterial diversity and microbiome complexity predict ecosystem functioning. Nat Commun. 2019;10:4841. 10.1038/s41467-019-12798-y. 31649246 PMC6813331

[72] Wang L, Liu ZL, Bres C, Jin GZ, Fanin N. Coniferous tree species identity and leaf aging alter the composition of phyllosphere communities through changes in leaf traits. Microb Ecol. 2024;87:126. 10.1007/s00248-024-02440-w. 39382725 PMC11464569

[73] Wang LX, Chen MX, Lam PY, Dini-Andreote F, Dai L, Wei Z. Multifaceted roles of flavonoids mediating plant-microbe interactions. Microbiome. 2022;10:233. 10.1186/s40168-022-01420-x. 36527160 PMC9756786

[74] Wang S, Wang XB, Han XG, Deng Y. Higher precipitation strengthens the microbial interactions in semi-arid grassland soils. Glob Ecol Biogeogr. 2018;27:570–80. 10.1111/geb.12718.

[75] Wang XG, Wiegand T, Hao ZQ, Li BH, Ye J, Lin F. Species associations in an old-growth temperate forest in north-eastern China. J Ecol. 2010;98:674–86. 10.1111/j.1365-2745.2010.01644.x.

[76] Wang ZH, Jiang Y, Zhang MH, Chu CJ, Chen YF, Fang S, Jin GZ, Jiang MX, Lian JY, Li YP, Liu Y, Ma KP, Mi XC, Qiao XJ, Wang XH, Wang XG, Xu H, Ye WH, Zhu L, Zhu Y, He FL, Kembel SW. Diversity and biogeography of plant phyllosphere bacteria are governed by latitude-dependent mechanisms. New Phytol. 2023;240:1534–47. 10.1111/nph.19235. 37649282

[77] Wu H, Gao TH, Hu A, Wang JJ. Network complexity and stability of microbes enhanced by microplastic diversity. Environ Sci Technol. 2024;58:4334–45. 10.1021/acs.est.3c08704. 38382548

[78] Wu MH, Chen SY, Chen JW, Xue K, Chen SL, Wang XM, Chen T, Kang SC, Rui JP, Thies JE, Bardgett RD, Wang YF. Reduced microbial stability in the active layer is associated with carbon loss under alpine permafrost degradation. Proc Natl Acad Sci USA. 2021;118(e2025321118). 10.1073/pnas.2025321118. PMC8237688 34131077

[79] Xiong C, Singh BK, He JZ, Han YL, Li PP, Wan LH, Meng GZ, Liu SY, Wang JT, Wu CF, Ge AH, Zhang LM. Plant developmental stage drives the differentiation in ecological role of the maize microbiome. Microbiome. 2021a;9:171. 10.1186/s40168-021-01118-6. 34389047 PMC8364065

[80] Xiong C, Zhu YG, Wang JT, Singh B, Han LL, Shen JP, Li PP, Wang GB, Wu CF, Ge AH, Zhang LM, He JZ. Host selection shapes crop microbiome assembly and network complexity. New Phytol. 2021b;229:1091–104. 10.1111/nph.16890. 32852792

[81] Yuan MM, Guo X, Wu LW, Zhang Y, Xiao NJ, Ning DL, Shi Z, Zhou XS, Wu LY, Yang YF, Tiedje JM, Zhou JZ. Climate warming enhances microbial network complexity and stability. Nat Clim Change. 2021;11:343–U100. 10.1038/s41558-021-00989-9.

[82] Yusoff MZM, Hu AY, Feng CJ, Maeda T, Shirai Y, Hassan MA, Yu CP. Influence of pretreated activated sludge for electricity generation in microbial fuel cell application. Bioresour Technol. 2013;145:90–6. 10.1016/j.biortech.2013.03.003. 23566463

[83] Zeng Q, Hu HW, Ge AH, Xiong C, Zhai CC, Duan GL, Han LL, Huang SY, Zhang LM. Plant-microbiome interactions and their impacts on plant adaptation to climate change. J Integr Plant Biol. 2025;67:826–44. 10.1111/jipb.13863. 39981843

[84] Zhang HJ, Chen WL, Dong LZ, Wang W. Grassland degradation amplifies the negative effect of nitrogen enrichment on soil microbial community stability. Glob Change Biol. 2024;30:3. 10.1111/gcb.17217. 38456565

[85] Zhang ZQ, Lu YH, Wei GH, Jiao S. (2022). Rare species-driven diversity–ecosystem multifunctionality relationships are promoted by stochastic community assembly. mBio, 13, 00449 – 22. 10.1128/mbio.00449-22. PMC9239226 35420485

[86] Zhou JZ, Deng Y, Luo F, He ZL, Yang YF. (2011). Phylogenetic molecular ecological network of soil microbial communities in response to elevated CO_2_. mBio, 2, e00122-11. 10.1128/mBio.00122-11. PMC3143843 21791581

