# Supplementary material for: Balancing deterministic and stochastic assembly during needle aging shapes phyllosphere microbial community complexity and stability
Source: Environ Microbiome. 2026 Jul 1;21:111. doi: 10.1186/s40793-026-00922-7 (PMC13599075; doi:10.1186/s40793-026-00922-7)
Supplement: Supplementary file 1 [file 40793_2026_922_MOESM2_ESM.docx]

**Supporting Information**

**Appendix S2. Supplementary Table and Figures**

**Table S1** Needle traits across age cohorts.

|  | Young needles | Mature needles | Old needles |
| --- | --- | --- | --- |
| TC (mg/g) | 505.11 (4.73) | 508.46 (4.31) | 517.15 (4.26) |
| TN (mg/g) | 23.40 (0.19) | 23.63 (0.19) | 23.39 (0.23) |
| TP (mg/g) | 1.47 (0.014) a | 1.35 (0.013) b | 1.33 (0.014) b |
| TC:TN | 21.72 (0.29) | 21.67 (0.29) | 22.30 (0.30) |
| TC:TP | 345.15 (4.67) c | 378.67 (4.84) b | 391.49 (5.32) a |
| TN:TP | 16.02 (0.23) b | 17.63 (0.24) a | 17.74 (0.27) a |
| Tannins (mg/g) | 15.30 (0.53) b | 22.24 (0.78) a | 22.71 (0.70) a |
| Flavonoids (mg/g) | 216.74 (12.01) c | 331.36 (14.96) b | 363.29 (15.80) a |

Values are represented as the mean value followed by standard error in parentheses. Different lowercase letters indicate significant differences at *p* < 0.05 when accounting for the random effects of tree species, sampling site, and individual identity. TC, total carbon; TN, total nitrogen; TP, total phosphorus.

**Fig. S1** Location of sampling sites. SS, Shengshan; FL, Fenglin; LS, Liangshui; ML, Muling; CBS, Changbaishan.

**Fig. S2** Rarefaction curves of bacterial (a) and fungal (b) communities across different samples.

**Fig. S3** Dominant bacterial phyla (relative abundance > 1%). Different lowercase letters indicate significant differences at *p* < 0.05 when accounting for the random effects of tree species, sampling site, and individual identity.

**Fig. S4** Dominant fungal phyla (relative abundance > 1%) and functional guilds. Different lowercase letters indicate significant differences at *p* < 0.05 when accounting for the random effects of tree species, sampling site, and individual identity.

**Fig. S5** β-nearest taxon index (βNTI) of bacterial and fungal communities across different tree species (a). Relative contributions of deterministic and stochastic processes to bacterial and fungal communities (b). DL, dispersal limitation; HeS, heterogeneous selection; HoS, homogeneous selection; HD, homogenizing dispersal; ED, ecological drift. Different lowercase letters indicate significant differences at *p* < 0.05.

**Fig. S****6** Multiple network features used to describe the network complexity of bacterial (a) and fungal (b) communities. Different lowercase letters indicate significant differences at *p* < 0.05 when accounting for the random effects of tree species, sampling site, and individual identity.

**Fig. S7** Absolute values of negative cohesion (i.e., negative species interactions) and positive cohesion (i.e., positive species interactions) of bacterial (a) and fungal (b) networks. Different lowercase letters indicate significant differences at *p* < 0.05 when accounting for the random effects of tree species, sampling site, and individual identity.

**Fig. S8** The relative effects of needle traits on multiple network stability indices of bacterial (a) and fungal (b) communities. **p* < 0.05 and ***p* < 0.01.
